# Ecological Niche Modeling of Risk Factors for H7N9 Human Infection in China

**DOI:** 10.3390/ijerph13060600

**Published:** 2016-06-16

**Authors:** Min Xu, Chunxiang Cao, Qun Li, Peng Jia, Jian Zhao

**Affiliations:** 1 State Key Laboratory of Remote Sensing Science, Institute of Remote Sensing and Digital Earth, Chinese Academy of Sciences, Beijing 100101, China; xumin@radi.ac.cn; 2 Public Health Emergency Center, Chinese Center for Disease Control and Prevention, Beijing 102206, China; fyklq@vip.sina.com (Q.L.); zhaoj1986@163.com (J.Z.); 3 Faculty of Geo-Information Science and Earth Observation (ITC), University of Twente, Enschede 7500, The Netherlands; jiapengff@hotmail.com; 4 Department of Epidemiology and Environmental Health, University at Buffalo, Buffalo, NY 14214, USA

**Keywords:** avian flu, H7N9, environmental factors, spatial modeling, MaxEnt

## Abstract

China was attacked by a serious influenza A (H7N9) virus in 2013. The first human infection case was confirmed in Shanghai City and soon spread across most of eastern China. Using the methods of Geographic Information Systems (GIS) and ecological niche modeling (ENM), this research quantitatively analyzed the relationships between the H7N9 occurrence and the main environmental factors, including meteorological variables, human population density, bird migratory routes, wetland distribution, and live poultry farms, markets, and processing factories. Based on these relationships the probability of the presence of H7N9 was predicted. Results indicated that the distribution of live poultry processing factories, farms, and human population density were the top three most important determinants of the H7N9 human infection. The relative contributions to the model of live poultry processing factories, farms and human population density were 39.9%, 17.7% and 17.7%, respectively, while the maximum temperature of the warmest month and mean relative humidity had nearly no contribution to the model. The paper has developed an ecological niche model (ENM) that predicts the spatial distribution of H7N9 cases in China using environmental variables. The area under the curve (AUC) values of the model were greater than 0.9 (0.992 for the training samples and 0.961 for the test data). The findings indicated that most of the high risk areas were distributed in the Yangtze River Delta. These findings have important significance for the Chinese government to enhance the environmental surveillance at multiple human poultry interfaces in the high risk area.

## 1. Introduction

A previously unidentified influenza A (H7N9) virus was first identified in a human in Shanghai, China, in February 2013. It spread out across most of eastern China soon after the first case, and subsequently became a new influenza pandemic [1,2]. The number of confirmed H7N9 human infection cases in China has reached 120 and it resulted in 21 deaths by 18 April 2013, which drew intensive attention from the local health authorities. The treatment of patients, field epidemiological investigation, specimen collection and inspection, medical observation of the close contacts, and enhancement of surveillance for the pneumonia cases of unknown origin have already being carried out. After receiving the case reports and the samples collected from patients, the China Center for Disease Control and Prevention (CDC) carried out a risk assessment for the potential spread of H7N9. The laboratory identification and confirmation were performed in a timely manner. Genetic characteristics of the newly identified virus in humans are currently being analyzed to determine potential factors that can explain the transmission from birds to humans. In China, a vast land spanning many degrees of latitude with complicated terrain, the climate varies radically. Many cities in the southeast, such as Shanghai, gradually closed all live-poultry markets after 4 April 2013 in order to control the H7N9 epidemic.

As the H7N9 virus causes no disease or only mild disease in birds [3,4], the virus may possibly spread silently among birds or other animal reservoirs without signs for the early warning of human infections, such as bird or poultry deaths [5]. It remains unclear how the H7N9 virus has been spread, and what roles different environmental risk factors may play in the pathways of transmission [5,6]. Specific prevention and control strategies have thus been difficult to develop. Although existing viruses such as H5N1 have been studied and kept under surveillance in China, it is important to identify the unique environmental risk factors that may be associated with presence of the H7N9 human cases.

Phylogenetic analysis has indicated that the H7 and N9 genes of the H7N9 virus might originate from H7N3 viruses in ducks in Zhejiang Province, China, and H7N9 viruses in the migratory birds, respectively [7]. Although much literature has revealed the epidemiological features and general risks of disease outbreak of H7N9 [7,8,9], no evidence of person-to-person transmission has been found. Some preliminary findings indicated that the outbreak and transmission of H7N9 may correlate with the migration of wild birds, the poultry trade, and the climate [7,10,11]. Previous research has demonstrated that influenza virus transmission was related to some climatic factors, including temperature, rainfall, and humidity [12,13,14,15]. Temporal distribution of the risks of H7N9 infection in Shanghai during 19 February–14 April 2013 showed that the H7N9 incidence rate was significantly associated with fortnightly mean temperature and rainfall [14]. Both daily minimum and maximum temperature may significantly contribute to human infection with the H7N9 virus. The models incorporating non-linear effects of minimum or maximum temperature on day 13 prior to disease onset were found to have the best predictive ability [15]. In addition, live poultry markets (LPMs), in which the virus was detected, may have played a critical role in the spread of H7N9 [16], despite a lack of poultry showing obvious symptoms of diseases.

The goal of this study is to examine the correlation between H7N9 human infection and environmental factors, predict the potential distribution of H7N9 among humans in China, and reveal the main meteorological and poultry-related risk factors of H7N9 infection. Our results show that (1) the distances to live poultry processing factories and to live poultry farms, and human population density were the three most important variables correlated with H7N9 diseases; and (2) the Yangtze River Delta is the highest risk area for H7N9 in China.

## 2. Materials and Methods

### 2.1. Data Source and Processing

#### 2.1.1. Disease Data

Data of H7N9 human cases were collected from the Public Health Emergency Center, China CDC. The data set includes the records of all 120 confirmed H7N9 human cases from the onset of the outbreak on 16 February to 17 April 2013. Each record contains the patient’s home address. Each human case was geocoded based on the latitude and longitude of his/her home address, which was assumed to represent the location of outbreak.

#### 2.1.2. Meteorological Data

This study selected several meteorological variables, such as temperature, precipitation and relative humidity, according to previous researches [7,17]. Epidemiological analyses, spurred by experimental data on influenza virus transmission and stability, have identified humidity, temperature and precipitation as climatic predictors of influenza epidemics in temperate regions of the world [18,19]. Influenza viruses are known to be more stable in the cold; thus, robust transmission at 5 °C and highly inefficient transmission at 30 °C may be due to an increased virus half-life at lower temperatures [18]. The 1 × 1 km monthly values of temperature and precipitation were available from WorldClim [19], which has been widely used in a variety of species distribution and disease pattern studies [20,21]. Six bioclimatic variables relating to the temperature and precipitation were selected for this study, including the annual mean temperature, maximum temperature of the warmest month, minimum temperature of the coldest month, annual precipitation, precipitation of the wettest month, and precipitation of the driest month. 

The annual relative humidity was acquired from the China Meteorological Data Sharing Service [22]. The original meteorological records of relative humidity were submitted and summarized based on 722 basic surface meteorological observation stations by administrative personnel in the meteorology departments of each province, city, and county. 

To acquire the visualized associations between the H7N9 disease and meteorological variables, the vector layer of H7N9 case distribution was overlapped with the vector layers of meteorological factors in the ArcGIS software (Version 9.3, ESRI Inc., Redlands, CA, USA). Each meteorological map was divided into five classes using equal interval method [23]. The method of the zonal statistics table in spatial analysis tools of ArcGIS software (Version 9.3, ESRI Inc.) was employed to extract the number of H7N9 cases from each of the different levels [12,24]. 

#### 2.1.3. Poultry Data

There are three types of poultry data which were related to H7N9 human infections, including live poultry farms, markets, and processing factories. The registered sites were extracted from the online directory from the State Bureau for Industrial and Commercial Administration (Mainland China). There were a total of 104 live poultry farms, 78 markets, and 630 processing factories registered in the system at the time of data collection from 16 February to 17 April 2013. Some small-scale stores and companies, without official registration or that were missed from the system due to not being updated, were also missed from our study. Three vector layers for poultry data were generated in ArcGIS (Version 9.3, ESRI Inc.). In order to transform the vector layers into raster layers, we use the method of calculating Euclidean distance to the special locations (namely live poultry farms, live poultry markets and live poultry markets) in the ArcGIS software (Version 9.3, ESRI Inc.) and generated the raster layers named “distances to live poultry farms”, “distances to live poultry markets” and “distances to live poultry processing factories”, namely the nearest distance to live poultry farms, live poultry markets and live poultry processing factories for each raster unit [25,26].

#### 2.1.4. Migratory Wild Bird Data

Migratory wild birds such as plovers, sandpipers, stints, curlews, and snipes make round-trip migrations of up to 26,000 km each year between their breeding grounds (northern hemisphere) and their non-breeding areas (southern hemisphere). Bird migration is considered to be of importance for predicting the transmission of avian influenza [10]. By large, there are three global migratory routes that pass through China, including the East Asian-Australasian flyway, the Black Sea-Mediterranean flyway, and the East Africa-West Asia flyway [27]. It is believed that the East Asian-Australia migration route is the most probable transmission route of H7N9, as the human infection rate of H7N9 along this migratory route has been higher than that of other peripheral regions [27]. Most of the H7N9 case outbreaks in eastern China were located close to the East Asian-Australasian flyway. Thus, we extracted the vector layer of the East Asian-Australasian flyway to represent the bird migration data that correlated to the H7N9 disease. 

Migratory wild birds tend to stop over in certain geographical areas, which are mainly wetlands that can provide abundant food for them and thus become particularly important in the context of the spread of disease. We included the wetland distribution as an important variable in the analysis, which was obtained from the State Key Laboratory of Remote Sensing Sciences, with a spatial resolution of 30 meters [28]. Extracted from Landsat Enhanced Thematic Mapper Plus (ETM+) images from 1999 to 2002, covering all of China, the composite images for wetland mapping were produced by combining the Thematic Mapper (TM) band-5 (Short Wave Infrared 1.55–1.75 um), band-4 (Near Infrared 0.79–0.90 um), and band-3 (Visible-Red 0.63–0.69 um) [29]. We generated a layer named “distance to the nearest wetland” based on the data of wetland distribution by calculating the Euclidean distance to the feature of interest in the ArcGIS software (Version 9.3, ESRI Inc.).

#### 2.1.5. Human Population Density Data

The population density in China is unevenly distributed, with the southeast region having a high population density while the northwest region has a low density. Moreover, even the population distribution within the same region is not homogeneous and has increased in recent years in China. We extracted the population data of China from the WorldPop dataset [30]. The WorldPop project was initiated in 2013 to unite the continent-focused AfriPop, AsiaPop and AmeriPop projects, with an aim of producing detailed and freely available population distribution and composition maps for the whole of Central and South America, Africa and Asia.

### 2.2. The Geography Statistic

To explore the potential breeding ground for the H7N9 virus to circulate, we overlapped the map of confirmed human cases onto the maps of the variables possibly contributing to the occurrence of human H7N9 infection. Spatial analytic approaches in the ArcToolbox in ArcGIS 9.2 software (ESRI Inc., Redlands, CA, USA) were employed to extract the number of H7N9 cases in different levels.

### 2.3. Ecological Niche Model

An ecological niche is a general term for the environment of various species and their living habitats [31,32]. It was first used to predict the potential geographical distribution of the species, and was proven to be effective for predicting the potential distribution of ecology- or environment-related diseases [12]. The most widely used Ecological Niche Modeling (ENM) methods include MaxEnt and GARP. MaxEnt is a general-purpose method for characterizing probability distribution from incomplete information based on the principle of maximum entropy. It outputs the maximum entropy distribution that satisfies a set of given environmental constraints. In place of true absences, MaxEnt uses background points to evaluate commission [33].

The model was run with the support of MaxEnt software (Version 3.3.1k, the MaxEnt and Bayesian Association of Australia, Inc., Canberra, Australia). Prediction for H7N9 potential spatial distribution based on environmental factors was meant to estimate the probability of the epidemic outbreak in unknown areas, according to spatial distribution and risk factors of known samples. We used the location of H7N9 cases in China between February and April 2013 as the known distribution data in MaxEnt. In order to avoid the bias of single random sample partition which can produce completely different ecological niche models, we used the *k*-fold cross-validation function in MaxEnt to randomly partition occurrence data into 10 random subsets when the selected training sample changes [33]. The random partitions rather than a single one were used to assess the average behavior of the algorithms. The term *k* represented the number of sub-training sets. In our study, *k* was arbitrarily assigned to 10. Environmental variable inputs included temperature, relative humidity, population density, and distances to live poultry farms, live poultry markets, live poultry processing factories, wetlands, and to the East Africa-West Asia bird migratory route. There was no collinearity found between environmental layers. All the included layers were interpolated in the spatial resolution of 1 km. The file format was set for logistic output, which rendered a predicted probability of presence ranging between 0 and 1. To measure the relative contribution of each environmental variable to the predictive model, a jackknife manipulation was performed [34]. The output—the suitable degree distribution of the H7N9 virus in China—had a value ranging from 0–1. The suitable degree was defined as the niche-fitness in ENM. In this study, a higher value of suitable degree indicated a higher risk of H7N9 outbreak; the value 0 meant the area was not possible for a H7N9 outbreak while the value 1 meant the most risk of H7N9. We fit and tested our model 10 times. In each iteration, only one subset was used to evaluate the model, and the other nine subsets were used to fit the model. The reported values were the average of 10 replicates for representing the degree of suitability of H7N9 presence in China.

To obtain risk levels of H7N9 incidence in each region, we divided the whole country into four risk level areas—non-, low, medium, and high risk—according to the degree of suitability [35]. Areas with a degree of suitability ≤0.01, between 0.01–0.1, between 0.1–0.3, and ≥0.3 were considered as non-risk, low risk, medium risk, and high risk areas, respectively [34].

The receiver-operating characteristic (ROC) curve was employed to evaluate the accuracy of the prediction model [36,37]. As a rule of thumb, generally speaking, when the area under the curve (AUC) value was between 0.5–0.7, the accuracy of the predictive model was considered to be relatively low; an AUC value between 0.7–0.9 and ≥0.9 indicated moderate and relatively high accuracy, respectively [35]. 

## 3. Results

### 3.1. The Relationship between Environmental Factors and H7N9 Diseases

As the results of spatial statistics using ArcGIS show, there were 109 cases distributed in the regions where the mean temperature from February to April was between −5 °C and 5 °C. Nine cases were distributed in the regions where the mean temperature was between −15 °C and −5 °C, and one case was located where the mean temperature was between 5 °C and 15 °C. There were no cases in regions where the mean temperature was above 15 °C or below −15 °C. Meanwhile, there were 27, 48, and 46 H7N9 cases distributed in regions where the mean monthly relative humidity ranged from 25%–45%, from 45%–65%, and from 65%–85%, respectively. No cases were distributed in regions where the mean relative humidity was less than 25% or more than 85%.

The distance to the bird migratory routes for 118 H7N9 cases (99% of all cases) was within 200 km of a bird migratory route; 100 of those cases were within 100 km, and 18 cases were between 100 and 200 km. Only one case was distributed in a region beyond 200 km from the bird migratory routes, located between 300 and 400 km. There were 118 cases of H7N9 within 100 km of a live poultry farm, 114 cases within 100 km of a live poultry processing factory, and 113 cases within 100 km of a live poultry market. Only several cases were distributed beyond 100 km from any of the three types of poultry locations. There were 116 H7N9 cases (97% of all cases) distributed in regions that were within 50 km from the nearest wetland. Three cases had a distance between 50 and 100 km from the nearest wetland. No cases were distributed in regions exceeding 100 km from the wetland.

### 3.2. The Potential Spatial Distribution Using MaxEnt

MaxEnt outputs are sensitive to the selection of background data. If the selection of background data points is from an area larger than the sampling extent it may result in a high AUC. Therefore, we restricted the selection of background data points to the sampling extent. In the case of the *k*-fold cross-validation procedure of MaxEnt, 10 different output predictions were generated based on default parameter values (0.01 convergence limit, and 1000 maximum iterations). The values reported were averaged over 10 replicates. 

**Figure 1:**
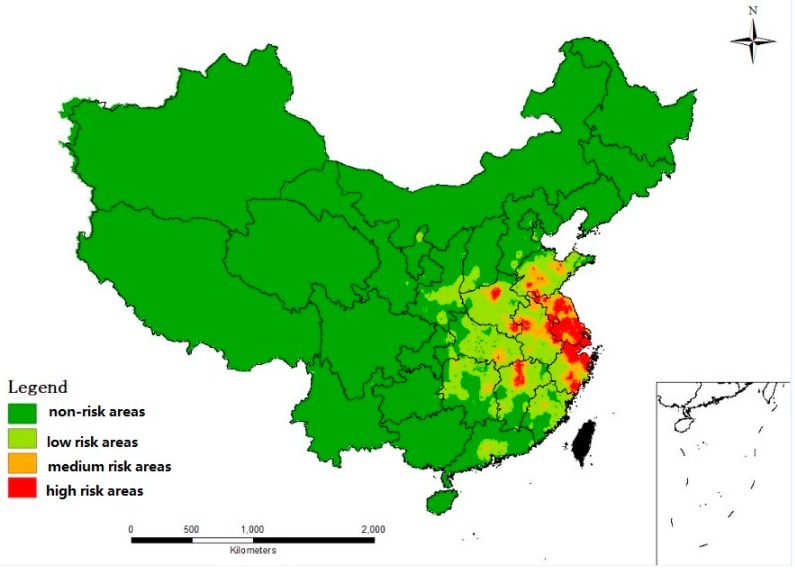
The map of potential spatial distribution of H7N9 risk in China. Areas with suitable degree values less than 0.01 were regarded as non-risk areas (green color), ranges from 0.01–0.1 were regarded as low risk areas (light green color); ranges from 0.1–0.3 indicated medium risk (orange color), and values over 0.3 indicated high risk (red color).

According to the partition, we developed a risk map of H7N9 in China, as shown in Figure 1. Southeast China had a relatively high suitable degree of H7N9. Some regions in central China had a relatively low suitable degree of H7N9. However, there was a very low suitable degree of H7N9 in the northeast and northwest China. Most regions in the Yangtze River Delta (such as Shanghai, Jiangsu) were found to be high risk areas for H7N9. However, the northwest regions and northeast regions that account for the majority of China appeared to be non-risk or low risk areas. 

**Figure 2:**
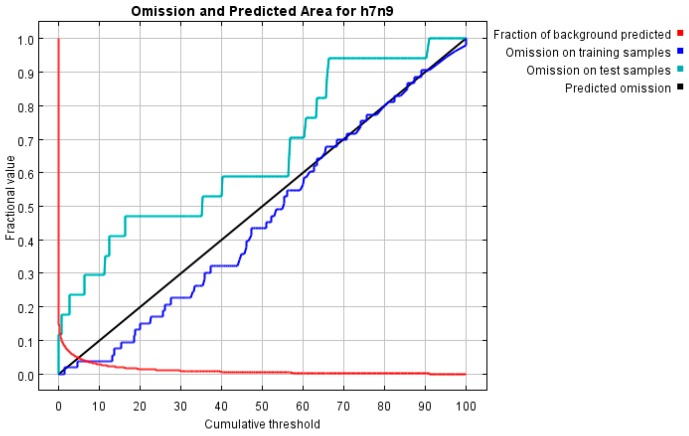
The omission rate and predicted area.

The AUC values were greater than 0.9 (0.992 for the training samples and 0.961 for the test data in average). Therefore, it is believed that this predictive model has a high accuracy. Figure 2 shows the omission rate and predicted area as a function of the cumulative threshold.

**Figure 3:**
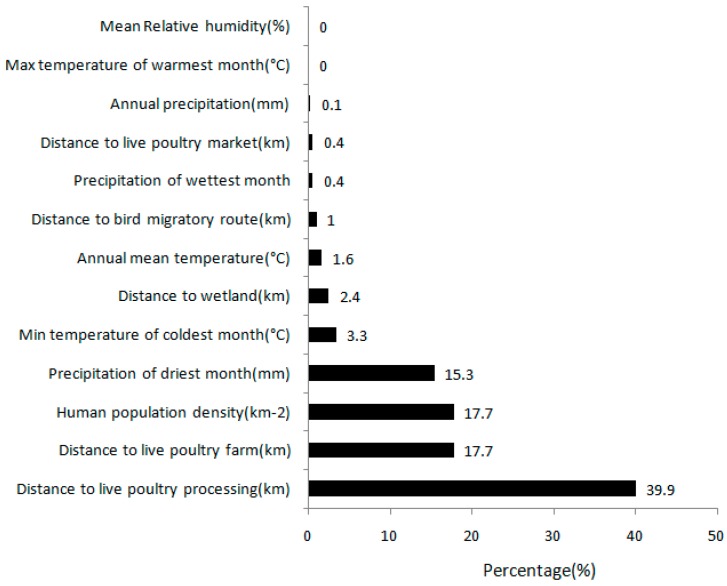
Percent contribution of each variable in the model.

The percent contribution of each environmental factor to the H7N9 predictive model is shown in Figure 3. The values reported are also averaged over 10 replicates. The distances to live poultry processing factories, to live poultry farms, and the human population density were the three most important environmental variables considered in this analysis correlated with H7N9 diseases; relative contributions to the model were 39.9%, 17.7%, and 17.7%, respectively. These three environmental factors accounted for more than 75.3% of the environmental relative contributions. The most important climatic factor considered in this analysis was the precipitation of the driest month, which had a 15.3% contribution to the model. The layers following in sequence are the minimum temperature of the coldest month (3.3%), the distance to the wetland (2.4%), the annual mean temperature (1.6%), the distance to the bird migratory route (1%), the precipitation of the wettest month (0.4%), the distance to a live poultry market (0.45), and the annual precipitation (0.1%). The maximum temperature of warmest month and the mean relative humidity had nearly no contribution to the model.

**Figure 4:**
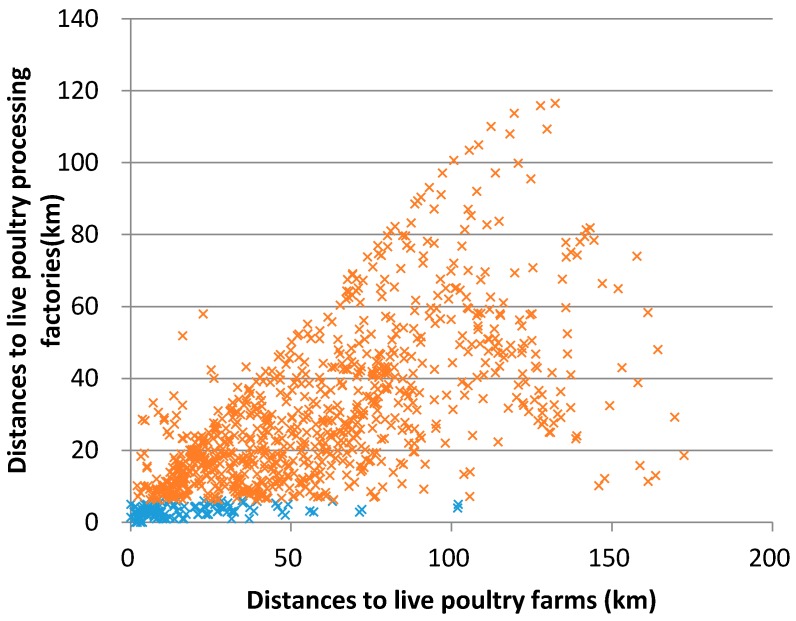
Modeled result of the ecological niche of H7N9 according to the distances to live poultry processing factories and distance to live poultry farms. The blue crosses represent the suitable ecological niche of H7N9.

**Figure 5:**
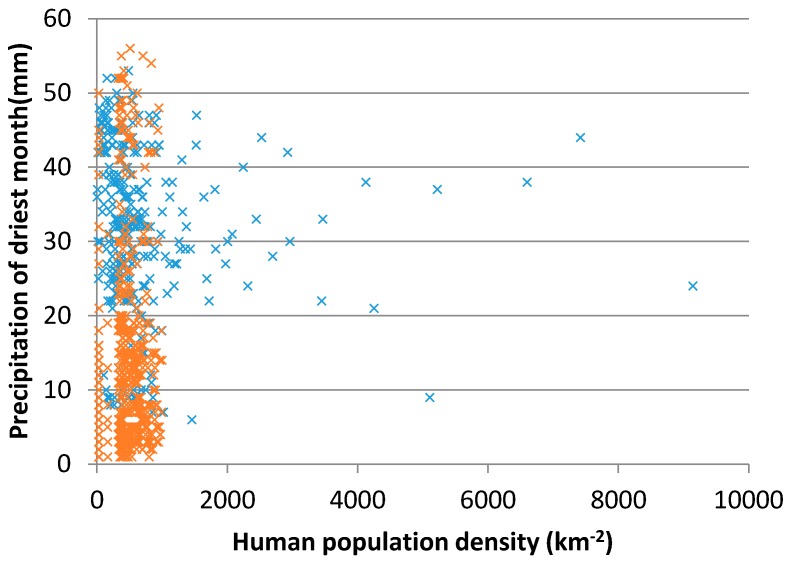
Modeled result of the ecological niche of H7N9 according to human population density and precipitation of driest month. The blue crosses represent the suitable ecological niche of H7N9.

We used scatter plots to show the impact of the significant environmental factors and the ecological niche variation of H7N9 [38]. Most of the H7N9 human infections were distributed in regions in which the distance to live poultry processing factories is within 5 km, and in areas in which the distance to live poultry farms is within 100 km (Figure 4). Furthermore, the area should have a high human population density and the precipitation of the driest month is between 20 and 50 mm (Figure 5). 

## 4. Discussion

We employed MaxEnt to predict the potential distribution of H7N9 in humans in China, for it has often shown accurate predictive capabilities in simulations and evaluations with only presence data. It outperforms some classical modeling approaches, such as domain, bioclim, and logistic regression [39]. Although GARP makes predictions in a similar way based on presence data, as well as continuous and/or categorical environmental variables, MaxEnt has been proved more effective in cases with a small sample size, which is more adequate for H7N9 prediction. However, the AUC test can be extremely biased when using the MaxEnt method for large geographical areas, due to the spatial sorting bias [31]. To overcome this disadvantage, *k*-fold cross-validation was employed to assess the average behavior of the algorithm, which divided the samples into 10 random partitions rather than a single one.

Our findings indicate that higher risks of H7N9 infection may exist in areas that are closer to live poultry farms and processing factories. This observation suggests that these places may be contaminated by the H7N9 virus, and have a high likelihood of occurrence of H7N9 infection in humans. Those residents who live or work at or near these places are more likely to be poultry raisers, processers, or consumers. These individuals have a disproportionately higher risk of contacting poultry and acquiring H7N9 infection. These findings indicate that the transmission of the H7N9 virus is naturally linked to exposure to live poultry and live poultry farms [16,38,40]. 

The precipitation of the driest month and the minimum temperature of the coldest month also have some impacts on H7N9. We still did not make it clear why the precipitation of the driest month impacted H7N9 so much. Proximity to the wetlands and the bird migratory routes has little impact on H7N9, though it is generally believed these locations had influence on wild bird migration, the primary driving force of avian influenza A transmission (H7N9).

Our statistics showed that most of the high risk areas (including Shanghai, Jiangsu, and Zhejiang) were distributed in the Yangtze River Delta. The whole region of Shanghai was and is a high risk area. Shanghai was the location of the first H7N9 outbreak and still presently reflects high disease prevalence. Up to 57% of the areas in Jiangsu Province and 37.11% of the areas in Zhejiang were at high risk. The southeast provinces (including Anhui, Jiangxi, Henan, Shandong, and Hubei) also contained a few areas of high risk, but the ratios of areas at high risk within these provinces were all less than 10%. The reason for the H7N9 epidemic outbreak in the Yangtze River Delta in February may be that the local temperature is very close to the most suitable temperature for the survival of avian influenza A virus (H7N9); the suitable temperature for the influenza A (H7N9) virus is between −5 °C and 5 °C. Moreover, these provinces contain many live poultry processing factories and live poultry farms, thereby increasing the risk of resident contact with poultry contaminated by avian influenza A (H7N9). The precipitation of the driest month in these provinces is very low, which also increased the probability of influenza virus transmitting from poultry to human.

The study has some limitations in the methodological approach. Firstly, this study used 13 environmental variables to predict the potential distribution of H7N9 human infection. However, we did not make it clear which environmental variables impacted the H7N9 poultry infection and which ones impacted the H7N9 human infection. Secondly, the data of live poultry farms, markets, and processing factories were extracted from the online directory from the State Bureau for Industrial and Commercial Administration (Mainland China), which only contained the large-scale sites. Some small-scale stores and companies, those without official registration or those missing from the system, were also missed in our study. Thirdly, the bioclimatic variables represent annual trends, seasonality and extreme or limiting environmental factors. The current climate data are interpolations of observed data, representative of 1950–2000, while the human cases are from 2013. Finally, in response to the influenza A (H7N9) epidemic in humans, enhanced environmental surveillance at multiple human poultry interfaces including live poultry farms, live poultry processing factory and live poultry markets has been progressively implemented since April 2013 [17]. The local governments had closed some live poultry markets and farms in the epidemic areas during the epidemic periods, and consequently we did not consider these factors in our study.

## 5. Conclusions

The niche model—based on several key environmental factors—was designed to predict the suitable areas in China for the occurrence of avian influenza A (H7N9). Our findings indicate that there were differences in the potential spatial distribution of avian influenza A (H7N9) among geographical regions in China, mainly impacted by the distribution of live poultry farms and live poultry processing factories. Results showed that most regions in the Yangtze River Delta, such as Shanghai and Jiangsu, are high risk areas for H7N9. However, the northwest regions and northeast regions that account for the majority of China are either non-risk or low risk areas. This observation suggests that the H7N9 virus is prevalent only in specific geographic and climatic areas. The distributions of live poultry processing factories and live poultry farms as well as human population density were the three most important environmental variables correlated with the H7N9 virus.

## Abbreviations

The following abbreviations are used in this manuscript:

ENM	Ecological Niche Model
ROC	Receiver Operating Characteristic
AUC	Area Under the Curve
